# Ischemic stroke causes Parkinson’s disease-like pathology and symptoms in transgenic mice overexpressing alpha-synuclein

**DOI:** 10.1186/s40478-022-01327-6

**Published:** 2022-02-24

**Authors:** Stephanie Lohmann, Jessica Grigoletto, Maria Eugenia Bernis, Verena Pesch, Liang Ma, Sara Reithofer, Gültekin Tamgüney

**Affiliations:** 1 German Center for Neurodegenerative Diseases (DZNE), Venusberg-Campus 1, Gebäude 99, 53127 Bonn, Germany; 2 Institut für Biologische Informationsprozesse, Strukturbiochemie (IBI-7), Forschungszentrum Jülich GmbH, Wilhelm-Johnen-Straße, 52425 Jülich, Germany; 3 Institut für Physikalische Biologie, Heinrich-Heine-Universität Düsseldorf, Universitätsstraße 1, 40225 Düsseldorf, Germany

**Keywords:** Ischemic stroke, Ischemia, Stroke, Parkinson’s disease, Synucleinopathy, Alpha-synuclein

## Abstract

The etiology of Parkinson’s disease is poorly understood and is most commonly associated with advancing age, genetic predisposition, or environmental toxins. Epidemiological findings suggest that patients have a higher risk of developing Parkinson’s disease after ischemic stroke, but this potential causality lacks mechanistic evidence. We investigated the long-term effects of ischemic stroke on pathogenesis in hemizygous TgM83 mice, which express human α-synuclein with the familial A53T mutation without developing any neuropathology or signs of neurologic disease for more than 600 days. We induced transient focal ischemia by middle cerebral artery occlusion in 2-month-old TgM83^+/−^ mice and monitored their behavior and health status for up to 360 days post surgery. Groups of mice were sacrificed at 14, 30, 90, 180, and 360 days after surgery for neuropathological analysis of their brains. Motor deficits first appeared 6 months after focal ischemia and worsened until 12 months afterward. Immunohistochemical analysis revealed ischemia-induced neuronal loss in the infarct region and astrogliosis and microgliosis indicative of an inflammatory response, which was most pronounced at 14 days post surgery. Infarct volume and inflammation gradually decreased in size and severity until 180 days post surgery. Surprisingly, neuronal loss and inflammation were increased again by 360 days post surgery. These changes were accompanied by a continuous increase in α-synuclein aggregation, its neuronal deposition, and a late loss of dopaminergic neurons in the substantia nigra, which we detected at 360 days post surgery. Control animals that underwent sham surgery without middle cerebral artery occlusion showed no signs of disease or neuropathology. Our results establish a mechanistic link between ischemic stroke and Parkinson’s disease and provide an animal model for studying possible interventions.

**Supplementary Information:**

The online version contains supplementary material available at 10.1186/s40478-022-01327-6.

## Introduction

Stroke is the third most common cause of death after ischemic heart disease and neonatal disorders, causing 9% of all fatalities worldwide, and even silent strokes without any immediate clinical manifestation are observed in up to 28% of the population [14, 54]. Approximately 87% of all stroke cases are ischemic, and are associated with a risk that increases with age, as 18% of individuals over 45 years of age have already experienced symptoms of stroke [20, 56]. Ischemic stroke leads to oxygen deprivation in the affected brain tissue with a subsequent loss of physiological cell functions and often permanent tissue damage within seconds [15, 41]. On a molecular basis, ischemia induces a cascade of deleterious events in the ischemic core, including an imbalance of membrane homeostasis due to Ca^2+^-influx, glutamate and cytochrome c release, oxidative and endoplasmic reticulum stress, mitochondrial dysfunction, activation of caspases, and inflammatory responses [15, 41]. Ultimately, ischemic stroke results in neuronal death by enhanced autophagy, apoptosis, and necrosis [15, 41]. Reoxygenation of ischemic tissue during reperfusion further enhances the detrimental primary effects of ischemia by an increase of neurotoxic reactive oxygen and nitrogen species [13, 21]. This secondary neuronal injury upon reperfusion also triggers inflammatory and pathological changes in brain regions connected to the ischemic core [8, 59]. Next to these short-term effects, ischemic stroke has many long-term consequences [56]. Stroke and silent strokes are associated with a higher risk of progression to dementia and cognitive decline [1, 30, 55]. Epidemiological studies have also revealed an increased risk of Parkinson’s disease (PD) after ischemic stroke, but there is currently no mechanistic model that adequately explains this association [6, 28].

PD is a neurodegenerative disorder characterized by pathologic depositions of α-synuclein in neurons that are referred to as Lewy bodies and Lewy neurites [4, 23, 49]. Pathologic α-synuclein is neurotoxic and causes the loss of dopaminergic neurons in the substantia nigra, which eventually results in motor symptoms due to an insufficient supply of the striatum with dopamine [23]. α-Synuclein is a 140 amino acid long and, under physiological conditions, mostly unstructured and soluble protein, which has regulatory functions in synaptic vesicle recycling in presynaptic terminals [11]. Unfavorable cellular conditions can induce α-synuclein to adopt pathologic conformations and assemble into oligomers and fibrils [42, 46–48]. These pathologic aggregates of α-synuclein have the capacity to recruit and template misfolding of naïve α-synuclein, and to propagate within the nervous system in a prion-like manner [7, 9, 27, 31, 34, 52]. While genetic risk factors, including mutations affecting the *SNCA* gene encoding α-synuclein, explain approximately 16–36% of the heritable risk of PD, the etiology of idiopathic PD is not well understood with advancing age being the biggest risk factor [3, 23, 38].

Here we investigated the role of mild ischemic stroke in the pathogenesis of PD in TgM83^+/−^ mice overexpressing human α-synuclein with the familial A53T mutation [19]. These mice do not naturally develop neuropathology or signs of neurologic disease for more than 600 days unless they are challenged with α-synuclein fibrils [10, 32, 57]. We induced transient focal cerebral ischemia in young TgM83^+/−^ mice by middle cerebral artery occlusion (MCAO) and investigated the effects of ischemia for up to a year post ischemia. Sham-treated mice remained healthy throughout the entire duration of the experiment and exhibited no neuropathological changes. In contrast, MCAO induced a synucleinopathy in TgM83^+/−^ mice as defined by cerebral α-synuclein aggregation and deposition, neuroinflammation, loss of dopaminergic neurons in the substantia nigra, and motor deficits. Our results show that ischemia can trigger sustained α-synuclein aggregation, providing a mechanistic link that explains how ischemic stroke increases the risk for PD.

## Materials and methods

### Mouse husbandry

Mice were housed under standard conditions with a 12 h light/dark cycle with free access to food and water. B6;C3-Tg(*Prnp*-SNCA*A53T)83Vle/J mice (short: TgM83 mice, The Jackson Laboratory) overexpressing the A53T mutant of human α-synuclein under the prion promoter on a C57BL/6 background [9] were crossed to wild-type C57BL/6 J mice and their progeny were genotyped for the presence of the transgene.

### Middle cerebral artery occlusion

Six- to eight-week-old male and female TgM83^+/−^ mice were subjected to either focal cerebral ischemia by middle cerebral artery occlusion (MCAO, n = 10/group) or a sham control surgery (sham, n = 6/group). TgM83^+/−^ mice were anaesthetized with isoflurane and treated with carprofen as an analgesic, after which surgery was carried out on a heating pad to prevent hypothermia. A midline incision was made and the right common carotid artery (CCA) was carefully separated from surrounding tissue and the vagus nerve. A permanent ligation was made 4 mm proximal to the bifurcation, which splits the CCA into the external carotid artery (ECA) and the internal carotid artery (ICA) using 6/0 sutures (Feuerstein). The CCA was clipped proximal to the bifurcation and the ECA was also clipped with a vascular clamp to prevent backflow from the distal vasculature. A microincision into the CCA, distal to the permanent knot, was made with spring scissors to insert a silicon-coated filament (Doccol) into the right CAA. Following insertion, the clip at the bifurcation was removed and the filament gently guided 9 to 11 mm into the ICA until the filament tip occluded the middle cerebral artery (MCA). The filament was fixed in this position for 30 min with an additional suture directly distal to the microincision. After 30 min of MCAO, the suture was shortly opened to remove the filament and then directly closed with a permanent ligation to allow natural reperfusion. The vascular clamp was removed and the neck incision was sutured with 4/0 black silk (Himed). The mice were then transferred to a heated cage and constantly monitored until full recovery from surgery. Sham control mice underwent the same surgical procedure of ligation and microincision of the CCA except for insertion of a filament. Treated animals were monitored daily for health and three times weekly for signs of neurological disease, such as reduced grooming, ataxia, bradykinesia, lethargy, paraparesis, paralysis, and kyphosis. Mice were sacrificed at 14, 30, 90, 180, and 360 days post surgery and their brains analyzed.

### Behavioral analysis

Motor function and coordination were evaluated with a rotarod test at 90, 180, and 360 days post surgery. Mice were placed on a horizontal and accelerating (4 to 40 rpm) rotarod treadmill (Ugo Basile) for a maximum of 300 s. Each animal was tested four times with a 5 min rest period between each trial on a particular test day, and the mean value of the last three trials was taken as an animal’s performance on that day.

### Immunohistochemical analysis

For immunohistochemical analysis, mice were overdosed with ketamine/xylazine and transcardially perfused with 0.9% (w/v) saline followed by 10% (v/v) formalin neutral buffer solution (Sigma). After removal, brains were fixed overnight in 10% formalin neutral buffer solution. Formalin-fixed brains were dehydrated in a series of graded ethanol and xylene baths, and embedded in paraffin. Brains were cut into 6-μm-thick coronal sections using a RM2255 microtome (Leica), mounted on glass slides, dried overnight, and stored at 4 °C. Brain sections were first deparaffinized in xylene, and then rehydrated in a series of graded ethanol baths. For antigen-retrieval either 1-min incubation in 88% formic acid or heat-induced antigen-retrieval with citric buffer (pH 6.0) was used. Endogenous peroxidase activity in the tissue was inhibited by incubation with 5% (v/v) hydrogen peroxide solution for 30 min. Brain sections were blocked with 20% (v/v) normal goat serum and 1% (v/v) bovine serum albumin (BSA) in 0.5% (v/v) Triton X-100 in PBS for 1 h at room temperature. The primary antibody was diluted in 1% (v/v) normal goat serum, 1% (v/v) BSA, and 0.25% (v/v) Triton X-100 in PBS and incubated overnight at room temperature. Antibodies used in this study with corresponding dilutions and antigen-retrievals are listed in Additional file 1. After washing once with 0.25% (v/v) Triton X-100 in PBS and twice with PBS, tissue sections were incubated for 1 h with peroxidase-conjugated secondary antibody using the Mouse on Mouse or Vectastain ABC kit (Vector Lab) diluted in 1% (v/v) normal goat serum and 1% (v/v) BSA in PBS at room temperature. The last step was omitted for phospho-α-synuclein-staining with the biotinylated pSyn#64 antibody (Wako). After washing once with 0.25% (v/v) Triton X-100 in PBS and twice with PBS, peroxidase-positive structures were visualized by incubation with DAB (3–3′-diaminobenzidine, Vector Labs). The oxidation process was stopped with 3% (v/v) hydrogen peroxide solution. Tissue sections were counterstained with Mayer’s hematoxylin (Merck), coverslipped with Eukitt (Sigma), scanned with a slide scanner (AxioScan.Z1, Carl Zeiss) or with an Epi-Scope1-Apotome (Carl Zeiss), and analyzed using Fiji software.

### Immunofluorescence analysis

Paraffin-embedded tissues were cut into 6-µm-thick coronal sections, mounted on glass slides, deparaffinized, and rehydrated as indicated before. For antigen retrieval, slides were incubated in citrate buffer (pH 6.0). After cooling down and washing twice with PBS, autofluorescence of the tissue was quenched by incubation in CuSO_4_ for 120 min at room temperature [45]. The slides were blocked in 20% (v/v) normal goat serum, 1% (v/v) BSA, and 0.5% (v/v) Triton X-100 in PBS for 1 h at room temperature. Sections were then incubated with a primary antibody in 1% (v/v) normal goat serum, 1% (v/v) BSA, and 0.25% Triton X-100 in PBS overnight at room temperature. Antibodies used in this study with the corresponding dilutions are listed in Additional file 1. After washing once with 0.25% (v/v) Triton X-100 in PBS, and twice with PBS, sections were stained with corresponding Alexa Fluor 488-, Alexa Fluor 594-, or Alexa Fluor 647-conjugated (Thermo Fisher Scientific) secondary antibodies and the nuclear dye DAPI (4′,6-diamidino-2-phenylindole; Thermo Fisher Scientific) in 1% (v/v) normal goat serum, 1% (v/v) BSA, and PBS for 1 h at room temperature. Slides were washed three times, coverslipped with Fluoromount medium (Sigma) and visualized with an LSM700 confocal laser-scanning microscope (Carl Zeiss).

### Preparation of mouse brain homogenates for biochemical analyses

For biochemical analyses brains were separated into ipsi- and contralateral hemispheres, snap-frozen on dry ice, and stored at −80 °C. Brain samples were homogenized in ice-cold Ca^2+^- and Mg^2+^-free PBS (pH 7.4) in the presence of phosphatase and protease inhibitors (HALT inhibitor cocktail; Thermo Fisher Scientific) by two 30-s cycles at 6500 rpm in a Precellys 24-Dual homogenizer (Peqlab) to reach a final concentration of 10% (w/v). Total protein concentration was determined using the Pierce BCA Protein Assay Kit (Thermo Fischer Scientific).

### Quantification of oligomerized and aggregated α-synuclein by fluorescence resonance energy transfer (FRET)

Oligomers and aggregates of human α-synuclein in brain homogenates were quantified by fluorescence resonance energy transfer (FRET) using a commercially available kit (6FASYPEG, Cisbio). Briefly, triplicates of 10-µL samples with 1 µg total protein were prepared from 10% (w/v) ipsi- and contralateral brain homogenate in 1 × lysis buffer. Ten microliter of a pre-mixed antibody solution containing anti-h-α-Synuclein-d2 (acceptor) and anti-h-α-Synuclein-Tb-Cryptase (donor) were added to each sample. A negative control, Cryptase control and buffer control were prepared as suggested by the company. Twenty microliter of the final mix containing sample and antibody as well as the controls were transferred on a 384-well flat clear-bottom plate (Greiner Bio-One), covered with a plate sealer, and incubated for 20 h at room temperature. Fluorescence emission was measured at 665 nm for FRET-dependent acceptor fluorescence and at 620 nm for FRET-independent donor fluorescence on a CLARIOstar microplate reader (BMG Labtech). The ratio of both fluorescence emission values multiplied by 10,000 is directly proportional to the amount of human α-synuclein oligomers and aggregates in each sample.

### Quantification of total human α-synuclein by immunoassay

The concentration of total human α-synuclein in brain homogenates was quantified by a colorimetric horseradish peroxidase-linked immunoassay using a commercially available anti-α-synuclein ELISA kit (AS-55550-H, AnaSpec). Briefly, 10% ipsi- and contralateral brain homogenate was diluted 1:100,000 in dilution buffer, transferred on a 96-well strip plate in triplicates, and co-incubated with 1 µg/mL of the detection antibody at 4 °C overnight. After washing the plate six times, TMB substrate solution (3,3′,5,5′-tetramethylbenzidine) was added. The color reaction was stopped after an incubation period of 5 min at 37 °C by adding stop solution, and absorbance was measured at 450 nm on a FLUOstar microplate reader (BMG Labtech).

### Quantification of the infarct volume in histological sections

The infarct volume of each brain was measured under masked conditions. Areas of NeuN-positive staining were measured using Fiji in the ipsi- and contralateral hemispheres of three MCAO-treated animals for each time point and of eight sham-treated animals. To minimize potential error caused by edema, the infarct volume was calculated in relation to the contralateral hemisphere according to Swanson et al. (contralateral hemisphere minus ipsilateral non-infarct area divided by contralateral hemisphere) [51]. The total infarct volume was measured in coronal sections ranging from bregma 0.14 mm to −1.22 mm and presented as mean percentage of the contralateral hemisphere ± standard error (SEM).

### Quantification of neuroinflammation in histological sections

Gliosis within the penumbra region was quantified in coronal sections ranging from bregma 0.26 mm to −1.70 mm in three sham-treated and five MCAO-treated animals per group and time point. Quantification was performed under masked conditions. Ionized calcium-binding adapter molecule 1 (Iba1)-positive staining was quantified in two separate tissue sections with six separate one square-millimeter-sized areas for each ipsi- and contralateral side. Glial fibrillary acidic protein (GFAP)-positive staining was quantified in two separate tissue sections with three separate one square-millimeter-sized areas for each ipsi- and contralateral side. Digitalized images were converted to an 8-bit format and the lower and upper thresholds were set to 0 and 130, respectively. The lower and upper thresholds represent mean values of manually analyzed images for MCAO- and sham-treated animals. The area of Iba1- or GFAP-positive staining was measured in each image using a Fiji macro. Data are presented as mean percentages ± standard deviation (SD).

### Quantification of dopaminergic neurons in histological sections

Quantification of dopaminergic neurons was based on a previously described method [26]. Dopaminergic neurons characterized by visible nuclei after hematoxylin staining and by entire neuronal somas positive for tyrosine hydroxylase were quantified under masked conditions in three sham-treated and five MCAO-treated animals per time point. The number of tyrosine hydroxylase-positive cells was counted in both brain hemispheres in matching sets of coronal midbrain sections at −2.90 mm, −3.08 mm, −3.26 mm, and −3.44 mm relative to bregma, and the area of the substantia nigra was identified corresponding to areas depicted in figures 55 to 60 of a mouse brain atlas [16]. The data was summarized for each group and hemisphere as mean tyrosine hydroxylase-positive cells ± standard deviation (SD) per section.

### Quantification of phosphorylated α-synuclein in histological sections

For quantification of brain areas with staining for phosphorylated α-synuclein, coronal sections at bregma 0.74 mm, −1.70 mm, −2.92 mm, −4.84 mm, and −5.68 mm were used. Digitalized images were masked prior to analysis. Cells positive for phosphorylated α-synuclein were counted in each region in both brain hemispheres of four animals at 360 days after stroke, and averages of phosphorylated α-synuclein-positive cells per square millimeter per region were presented in a color-coded heat map.

### Statistical analysis

Statistical tests were carried out using Prism 8.0 (GraphPad). To assess differences among multiple groups two-way analysis of variance (ANOVA) with multiple comparisons was used, based on the row factor time point and the repeated measures in each column factor of ipsi-/ contralateral stroke or sham surgery. Due to the multiple comparisons of the row and column means, we corrected using Tukey’s post-hoc test. As the calculation of the infarct volume already included ipsi- and contralateral hemispheres to reduce the effect of the edema, one-way ANOVA was used to assess differences within the sham group and stroke group at the different time points. Data are presented as mean ± standard deviation (SD), unless stated otherwise. *P* values below 0.05 were considered statistically significant.

## Results

### TgM83^+/−^ mice subjected to MCAO continuously gain body weight but develop motor deficits within 180 days after surgery that further worsen over time

**Fig. 1:**
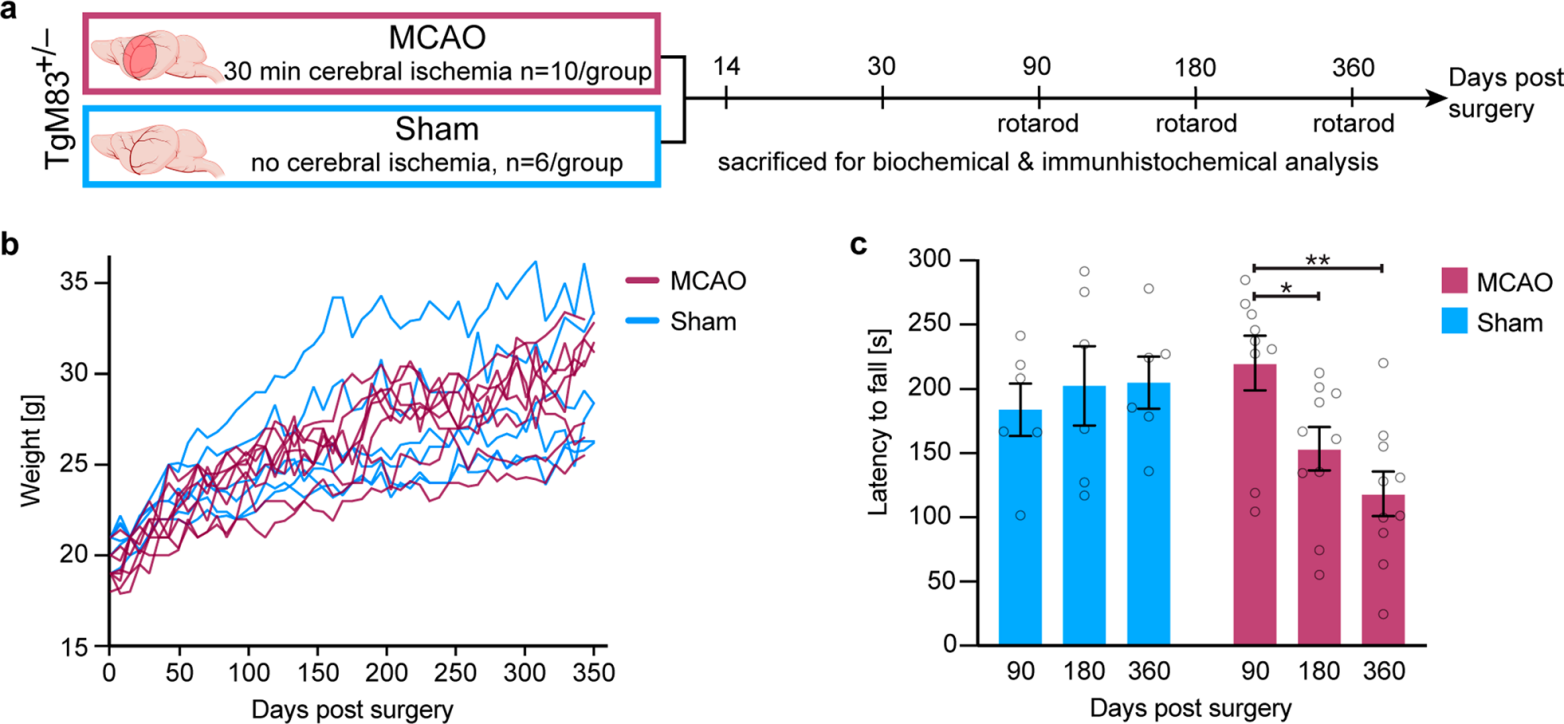
TgM83^+/−^ mice subjected to MCAO gain weight but develop motor deficits within 180 days. TgM83^+/−^ mice expressing the A53T mutant of human α-synuclein were subjected to middle cerebral artery occlusion (MCAO) for 30 min or sham surgery without occlusion of the artery. Groups of treated animals were sacrificed at 14, 30, 90, 180, and 360 days post surgery and neuropathology was analyzed by histology and biochemistry (**a**). Additionally, the motor behavior of treated animals was tested at 90, 180, and 360 days post surgery on a rotarod treadmill (**a**). TgM83^+/−^ mice subjected to MCAO (n = 8, red lines) as well as sham-treated animals (n = 6, blue lines) gained body weight until the end of the experiment at 360 days post surgery (**b**). MCAO- and sham-treated mice were tested for motor impairment using a rotarod at 90, 180, and 360 days post surgery. TgM83^+/−^ mice that had been subjected to MCAO (red) showed significantly reduced motor skills at 180 and 360 days post surgery relative to 90 days post surgery (**c**). In contrast, sham-treated control animals (blue) did not develop any motor deficits. Six to ten animals were analyzed per group. The data represents the mean latency to fall in seconds ± standard error of the mean. *P* values were computed using two-way ANOVA followed by Tukey’s post-hoc test (*P*: * < 0.05, ** < 0.01)

We induced a mild, 30-min-long focal ischemia in the right brain hemisphere of 6–8-week-old male and female TgM83^+/−^ mice by middle cerebral artery occlusion (MCAO). Control mice underwent sham surgery without occlusion of their right middle cerebral artery. All animals subjected to surgery quickly recovered within an hour without showing any lasting signs of disability or disease. Because we were only interested in the long-term effects of stroke, motor deficits were not tested until 90 days after stroke. Mild motor deficits that might have been present at earlier time points during the acute reperfusion phase were not examined. Groups of 10 treated animals were sacrificed 14, 30, 90, 180, and 360 days post surgery for histological and biochemical analyses of both brain hemispheres (Fig. 1a). At these predefined endpoints, none of the animals displayed any overt signs of neurological illness, such as kyphosis, ataxia, or loss in body weight, which TgM83^+/−^ mice develop given enough time after challenge with preformed fibrils of α-synuclein or brain homogenates of patients with multiple system atrophy containing α-synuclein aggregates [10, 32, 57]. In addition, TgM83^+/−^ mice subjected to MCAO as well as the control animals, which underwent surgery, had an overall gain in body weight until the end of the experiment at 360 days post surgery (Fig. 1b). Obvious differences between MCAO- and sham-treated animals only became detectable when tested for motor impairment using a rotarod treadmill 90, 180, and 360 days post surgery (Fig. 1c). In contrast to sham-treated control animals, which did not develop any motor deficits, TgM83^+/−^ mice that had undergone MCAO showed a significant 30% reduction in their motor skills 180 days post surgery that further dropped to 46% at 360 days post surgery compared to their performance at 90 days post surgery.

### TgM83^+/−^ mice display an early and a recurring late neuronal loss after stroke

**Fig. 2:**
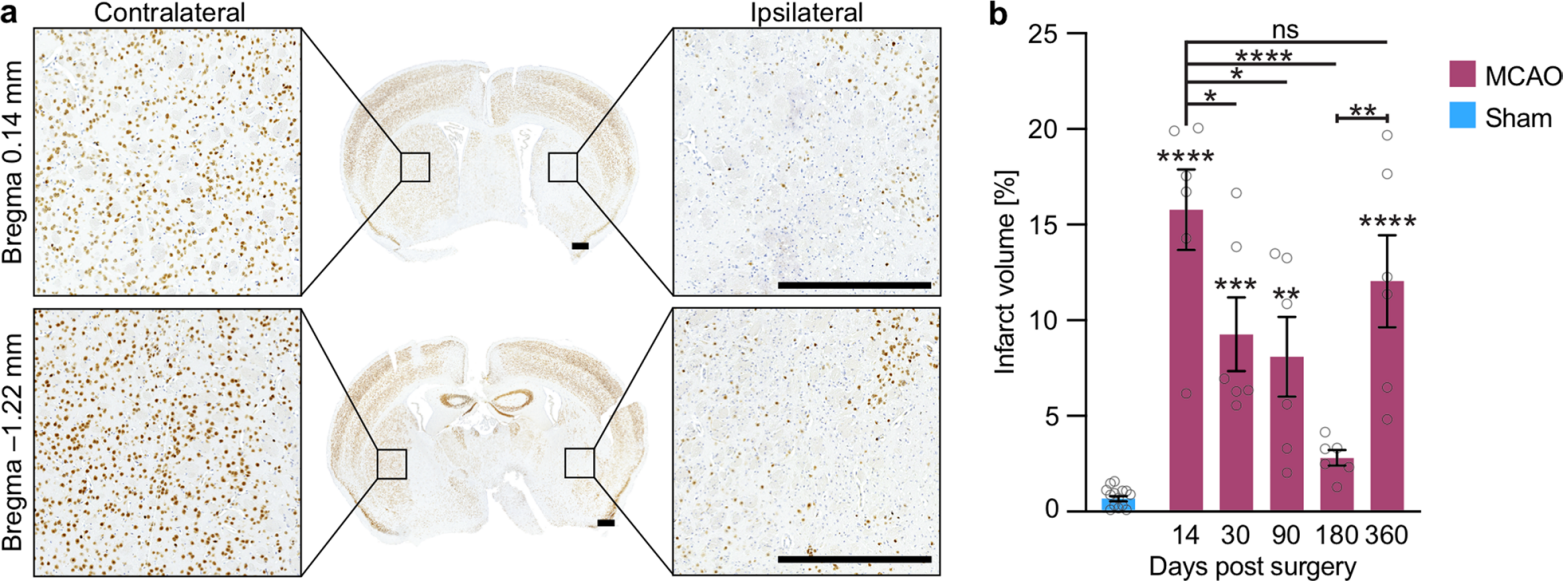
TgM83^+/−^ mice show neuronal loss in the ipsilateral brain hemisphere after MCAO. Immunohistochemical staining for the neuronal marker NeuN showed an ipsilateral loss of neurons in ischemic brains of TgM83^+/−^ mice after MCAO (**a**). Quantification of brain tissue sections ranging from bregma 0.14 mm to −1.22 mm of mice subjected to MCAO revealed significant ipsilateral neuronal degeneration in the ischemic core and peri-infarct region at 14, 30, 90, 180, and 360 days post surgery relative to sham-treated animals (**b**). The infarct size was calculated in relation to the contralateral brain hemisphere to minimize the error caused by edema. Two coronal brain sections of three MCAO-treated animals were analyzed for each time point and two coronal brain sections of eight sham-treated animals for all time points together. The data represents the mean infarct volume in percent ± standard error of the mean. *P* values were computed using one-way ANOVA followed by Tukey’s post-hoc test (*P*: **** < 0.0001, *** < 0.001, ** < 0.01). The scale bar represents 0.5 mm

To quantify the lesion volume in the brains of TgM83^+/−^ mice after MCAO, we prepared brain tissue sections and stained neurons by immunohistochemistry with the MAB377 antibody for NeuN (Fig. 2a). The brains of all stroke animals exhibited typical subcortical damage in the striatum (Fig. 2a), whereas brains of control animals did not show any structural changes (Additional file 2). At fourteen days after MCAO we measured an average infarct volume of 15.8% in the ipsilateral brain hemisphere relative to the healthy contralateral brain hemisphere of TgM83^+/−^ mice (Fig. 2b). By 180 days post surgery the original infarct volume had gradually decreased to 2.8% (Fig. 2b). Reductions in the lesion volume after mild cases of cerebral ischemia over extended periods of time have been reported previously [24, 43]. Surprisingly, by 360 days after MCAO the lesion volume had increased to 12.0% again, indicative of a renewed neurodegenerative process in the original lesion area beginning sometime between 180 and 360 days after surgery. In contrast, brain tissue sections of TgM83^+/−^ mice that had only undergone sham surgery revealed very little to no neuronal loss over the entire 360-day period after surgery (Fig. 2b, Additional file 2).

### TgM83^+/−^ mice display a biphasic inflammatory process in the brain after stroke

**Fig. 3:**
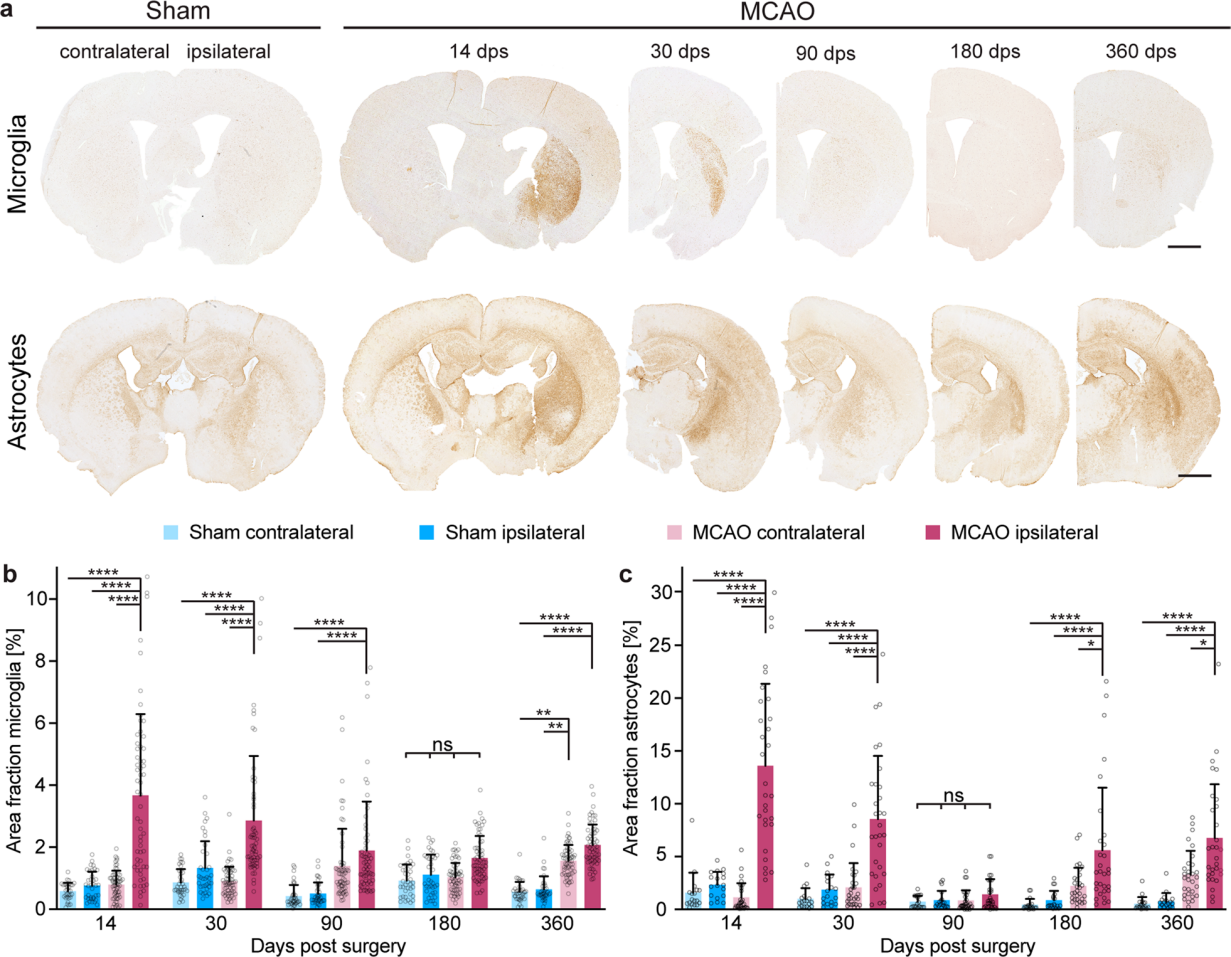
TgM83^+/−^ mice display early and late inflammatory responses in the brain after MCAO. Microglia and astrocytes in coronal brain tissue sections of MCAO- and sham-treated TgM83^+/−^ mice were stained with antibodies to Iba1 or to GFAP, respectively (**a**). Quantification of the stainings revealed that microgliosis (**b**) and astrogliosis (**c**) were significantly induced at 14 days post surgery within the stroke lesion area of mice subjected to MCAO (dark red) when compared to the contralateral hemisphere (light red), or the ipsi- (dark blue) and contralateral (light blue) brain hemispheres of sham-treated animals. Beginning at 30 days post surgery we measured a continuous decline in the level of inflammation, which was reduced to levels observed in sham-treated animals at 90 days post surgery for astrogliosis and later at 180 days post surgery for microgliosis. Following its initial clearance, the inflammatory reaction, surprisingly, began to resurface again in the brains of MCAO-treated animals. We measured a continuous rise of astrogliosis from 90 to 360 days post surgery in the ipsilateral brain hemisphere of MCAO-treated animals. At 360 days post surgery also microgliosis was significantly induced again in the ipsilateral and, importantly, also in the contralateral brain hemisphere of MCAO-treated animals. A continuous increase of astrogliosis was also measured in the contralateral brain hemisphere of MCAO-treated animals at 180 and 360 days post surgery but did not reach significance. The data represent the mean area fraction in percent ± standard deviation. Six to twelve coronal brain sections of three to five animals were analyzed per group at 14, 30, 180, and 360 days post surgery. *P* values were computed using two-way ANOVA followed by Tukey’s post-hoc test (*P*: * < 0.05, **** < 0.0001). Scale bars represent 1.0 mm

To assess neuroinflammatory changes in the brains of TgM83^+/−^ mice after MCAO and sham surgery, we prepared coronal brain tissue sections of animals sacrificed at 14, 30, 90, 180, and 360 days post surgery and stained astrocytes with an antibody to GFAP and microglia with an antibody to Iba-1 (Fig. 3a). Quantification of the GFAP- and Iba-1-staining in and around the lesion area revealed that following MCAO microgliosis (Fig. 3b) and astrogliosis (Fig. 3c) were significantly induced in the brains of mice subjected to MCAO at 14 days post surgery when compared to the contralateral hemisphere. The primary inflammatory reaction gradually subsided and astrogliosis had fully receded by 90 days and microgliosis by 180 days post surgery. In response to the secondary neurodegenerative process that we detected in the lesion area in the brains of mice between 180 and 360 after MCAO, astrogliosis was induced again sometime between 90 and 180 days post surgery followed by microgliosis after 180 days post surgery. Also, by 360 days post surgery, inflammation had spread from the ipsilateral to the contralateral brain hemisphere of MCAO-treated mice, as seen by significantly elevated levels of microgliosis in both brain hemispheres in comparison to sham-treated animals (Fig. 3, Additional file 3). A similar gradual increase of astrogliosis was also seen in the contralateral brain hemisphere of MCAO-treated animals at 180 and 360 days post surgery without reaching significance.

### TgM83^+/−^ mice display a late loss of dopaminergic neurons in the substantia nigra after stroke

**Fig. 4:**
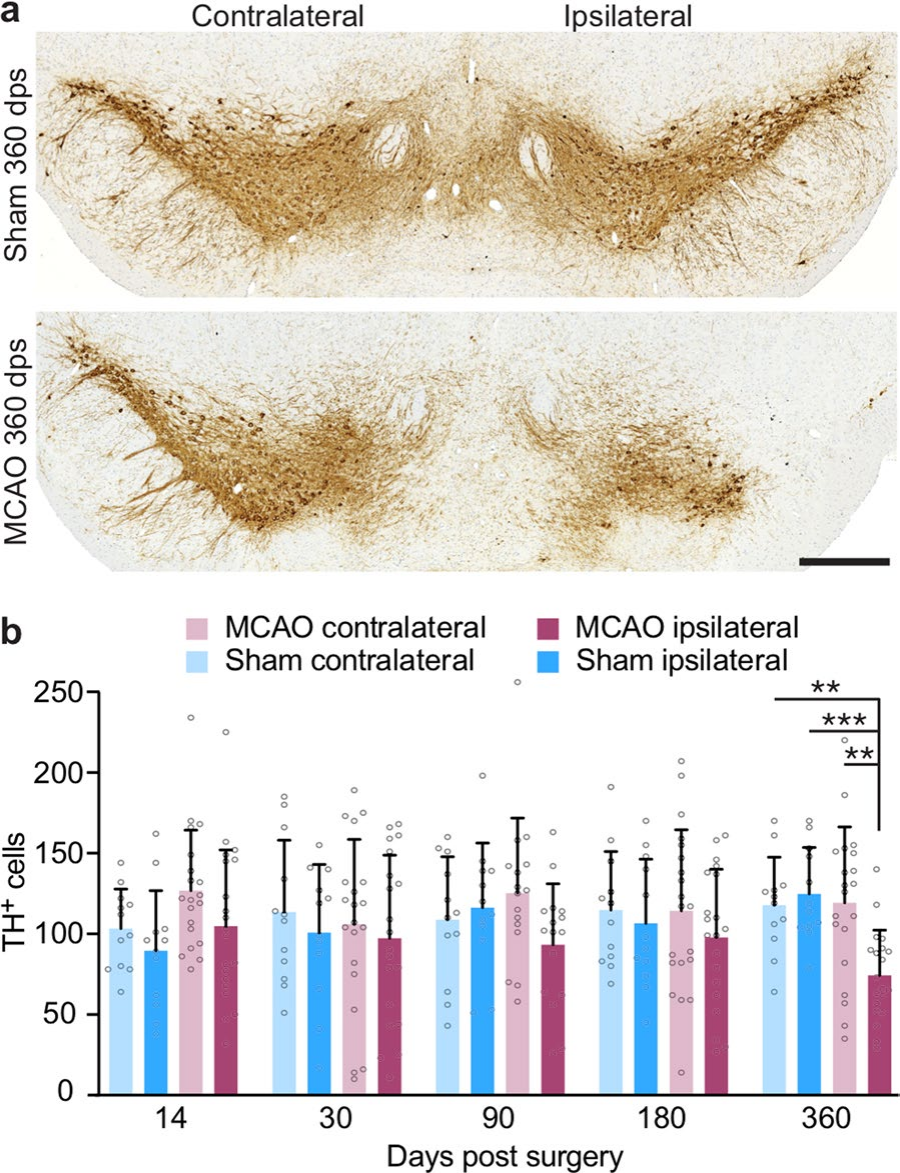
MCAO-treated TgM83^+/−^ mice display a late loss of dopaminergic neurons in the substantia nigra. Immunhistochemical analysis of the substantia nigra of MCAO- and sham-treated TgM83^+/−^ mice with an antibody against TH showed an ipsilateral loss of dopaminergic neurons 360 days after MCAO (**a**). Quantification of TH-positive cells revealed a significant loss of dopaminergic neurons on the ipsilateral side of the substantia nigra of TgM83^+/−^ mice after MCAO-treatment (dark red) relative to the contralateral side (light red) or to sham-treated control animals (dark and light blue) at 360 days post surgery (**b**). The data represent the mean count of TH-positive cells per coronal section of the substantia nigra ± standard deviation. Four coronal brain sections of three to five animals were analyzed per group 14, 30, 90, 180, and 360 days post surgery. *P* values were computed using two-way ANOVA followed by Tukey’s post-hoc test (*P*: ** < 0.01, *** < 0.001). The scale bar represents 0.5 mm

To investigate whether the motor deficits, which TgM83^+/−^ mice displayed beginning at 180 days after stroke, may have been caused by a loss of dopaminergic neurons in the substantia nigra, we stained midbrain tissue sections of animals sacrificed at 14, 30, 90, 180, and 360 days post surgery with an antibody to tyrosine hydroxylase (TH) (Fig. 4a). Quantification of tyrosine hydroxylase-positive neurons of the substantia nigra revealed that a significant number of dopaminergic neurons was lost by 360 days post surgery in the ipsilateral brain hemisphere of mice that had been subjected to MCAO (Fig. 4b). In contrast, no significant loss of dopaminergic neurons was detected in the contralateral brain hemisphere of mice subjected to MCAO, or in the brains of mice that had been subjected to sham surgery (Fig. 4b).

### Stroke induces aggregation of α-synuclein in the CNS of TgM83^+/−^ mice

**Fig. 5:**
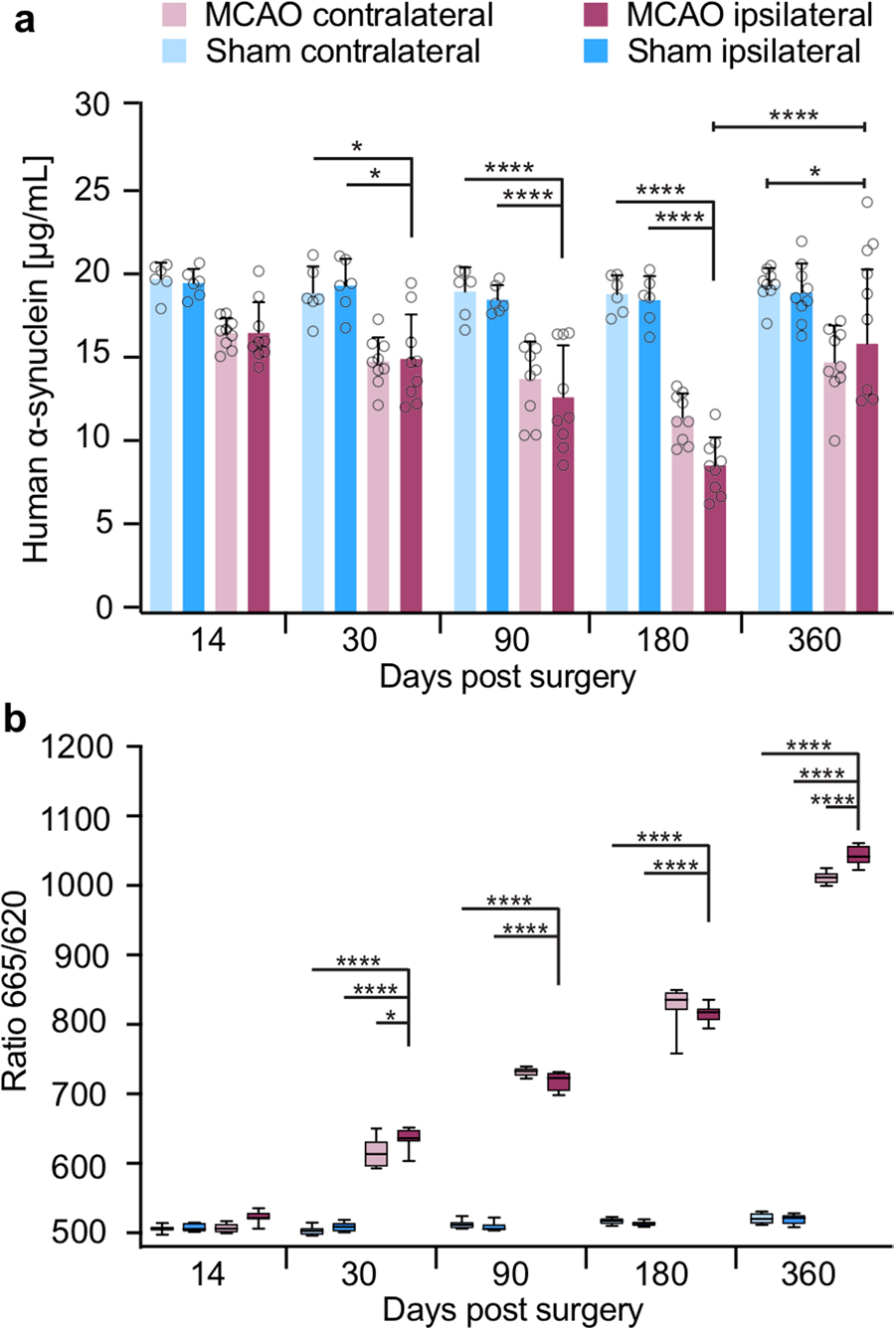
MCAO induces a continuous long-term increase of aggregated α-synuclein species in the CNS of TgM83^+/−^ mice. Quantification of the amount of human α-synuclein protein by ELISA in brain homogenates of TgM83^+/−^ mice subjected to MCAO showed a continuous decline in the ipsi- (dark red) and contralateral (light red) brain hemisphere up to 180 days post surgery, after which α-synuclein levels slightly rose again but did not reach levels detected in the brains of animals at 360 days post sham surgery (**a**). No changes in α-synuclein protein levels were observed in the ipsi- (dark blue) or contralateral (light blue) brain hemisphere of animals after sham surgery. Quantification of aggregated α-synuclein species by FRET analysis in brain homogenates of TgM83^+/−^ mice revealed a steady increase in the ipsi- (dark red) and contralateral (light red) brain hemisphere beginning 30 days post surgery (**b**). In contrast, α-synuclein did not aggregate in the brains of TgM83^+/−^ mice after sham surgery. Both brain hemispheres of three animals each were analyzed per group at 14, 30, 90, 180, and 360 days post surgery. The ELISA data represents the mean ± standard deviation. The FRET data are shown as boxplots. *P* values were computed using two-way ANOVA followed by Tukey’s post-hoc test (*P*: * < 0.05, **** < 0.0001)

To assess if the loss of dopaminergic neurons in the substantia nigra could be due to an accumulation of toxic species of aggregated α-synuclein, we first quantified the amount of human α-synuclein protein in the brains of TgM83^+/−^ mice after stroke by ELISA (Fig. 5a). Following MCAO, we observed an initial apparent decline in α-synuclein levels in both brain hemispheres of TgM83^+/−^ mice until 180 days post surgery, after which α-synuclein levels slightly rose again but remained below those measured in animals at 360 days after sham surgery (Fig. 5a). Sham surgery did not cause significant changes in α-synuclein protein levels at any time (Fig. 5a). Because oligomerization and aggregation of α-synuclein after stroke could explain an apparent reduction in α-synuclein protein levels, we next quantified the amount of aggregated α-synuclein in the brains of TgM83^+/−^ mice after stroke by FRET analysis (Fig. 5b). Surprisingly, we measured continuously rising levels of oligomerized/aggregated α-synuclein in both brain hemispheres between 14 and 360 days after stroke. In contrast, sham surgery did not induce detectable aggregation of α-synuclein at any time (Fig. 5b).

### Brains of symptomatic TgM83^+/−^ mice reveal intracellular deposits of pathologic α-synuclein

**Fig. 6:**
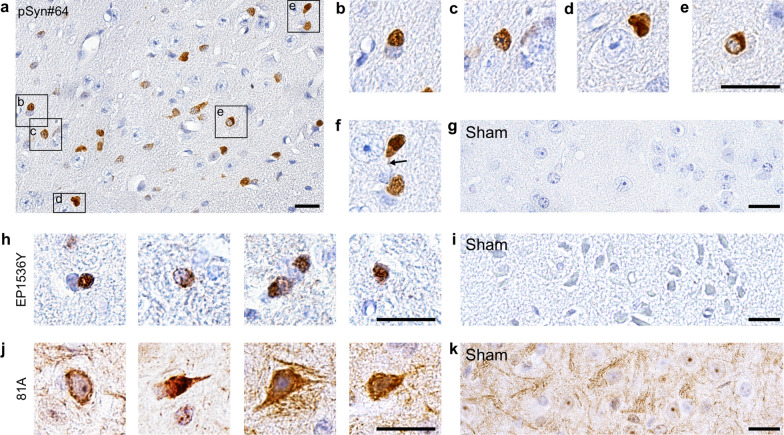
Diseased TgM83^+/−^ mice display various types of pathologic α-synuclein deposits in the CNS. Immunohistochemical staining of coronal brain tissues sections of diseased TgM83^+/−^ mice revealed abundant deposits of phosphorylated α-synuclein throughout the brain at 360 days after stroke using the pSyn#64 (**a–f**), EP1536Y (**h**), and 81A (**j**) antibodies that recognize phosphorylated α-synuclein at Ser129, which is associated with the accumulation of pathologic α-synuclein. Deposits of phosphorylated α-synuclein were mostly cytosolic (**b–d**), sometimes perinuclear (**e**), or dendritic (arrow) (**f**). In contrast, sham-treated mice did not show any deposits of phosphorylated α-synuclein with any of the three antibodies used (**g, i, k**). Staining with the 81A antibody resulted in a higher background due to a known cross reactivity of this antibody with phosphorylated neurofilament subunit L (**j, k**). The scale bars represent 20 µm

**Fig. 7:**
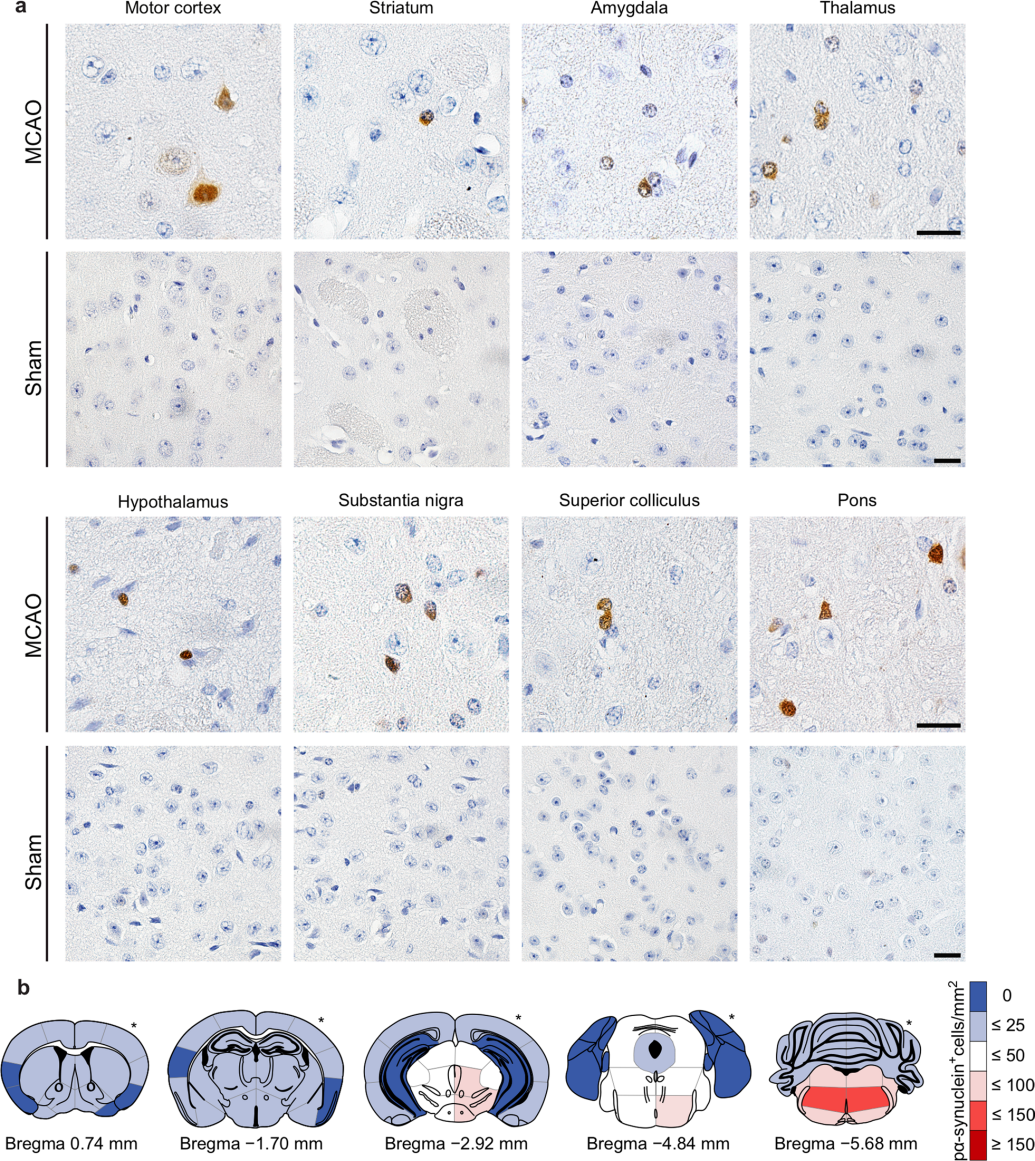
Diseased TgM83^+/−^ mice harbor deposits of pathologic α-synuclein in various brain regions. To reveal deposits of pathologic α-synuclein, coronal tissue sections of different brain regions of diseased TgM83^+/−^ mice collected at 360 days after stroke were stained with the pSyn#64 antibody against α-synuclein phosphorylated at serine 129. Diseased animals that had been subjected to stroke but not sham-treated animals showed cellular deposits of pathologic α-synuclein throughout the cerebrum, including the motor cortex, the striatum, the amygdala, the thalamus, the hypothalamus, the substantia nigra, the superior colliculus, and the pons (**a**). Deposits of pathologic α-synuclein in the brains of diseased TgM83^+/−^ mice at 360 days after MCAO were quantified as phosphorylated α-synuclein-positive cells per square millimeter from four diseased animals per brain region (Additional file 4), and the data were summarized in heat maps spanning five rostral-to-caudal brain areas (**b**). Asterisks indicate the stroke side. Scale bars represent 20 µm

To further assess α-synuclein aggregation in the CNS of TgM83^+/−^ mice after stroke, we stained coronal brain tissue sections of animals sacrificed at 360 days after MCAO or sham surgery by immunohistochemistry with three different antibodies, pSyn#64 (Fig. 6a–g), EP1536Y (Fig. 6h, i), and 81A (Fig. 6j, k), recognizing phosphorylated serine at position 129 of α-synuclein, which is associated with the cellular accumulation of pathologic α-synuclein deposits (Fig. 6) [17, 22, 33]. All three antibodies detected cytosolic, perinuclear, and dendritic accumulation of pathologic α-synuclein. Staining with the 81A antibody resulted in more background than with the other two antibodies as it also cross reacts with phosphorylated neurofilament subunit L [44]. In contrast, animals that had undergone sham surgery did not exhibit deposits of pathologic α-synuclein (Fig. 6g, i, k). We detected deposits of pathologic α-synuclein in various brain regions (Fig. 7a). A systematic quantification of brain pathology showed that deposits of phosphorylated α-synuclein were rarely present in rostral or cortical brain regions (bregma 0.74 mm and –1.70 mm). In contrast, 25–100 cells/mm^2^ positive for phosphorylated α-synuclein were detected in more caudal midbrain regions (bregma –2.92 mm and –4.84 mm), and with a trend for increased deposition in the ipsilateral versus the contralateral hemisphere (Fig. 7b, Additional file 4). Most pathology was localized in the brain stem (bregma –5.68 mm) with up to 150 cells/mm^2^ positive for phosphorylated α-synuclein in both hemispheres (Fig. 7b, Additional file 4). Pathology was most pronounced 360 days post surgery but cellular deposits of pathologic α-synuclein in some animals were also detectable already 180 days after stroke but not earlier (Additional file 5).

### Pathologic α-synuclein colocalizes with neurons, oligodendrocytes, and microglia in symptomatic TgM83^+/−^ mice

**Fig. 8:**
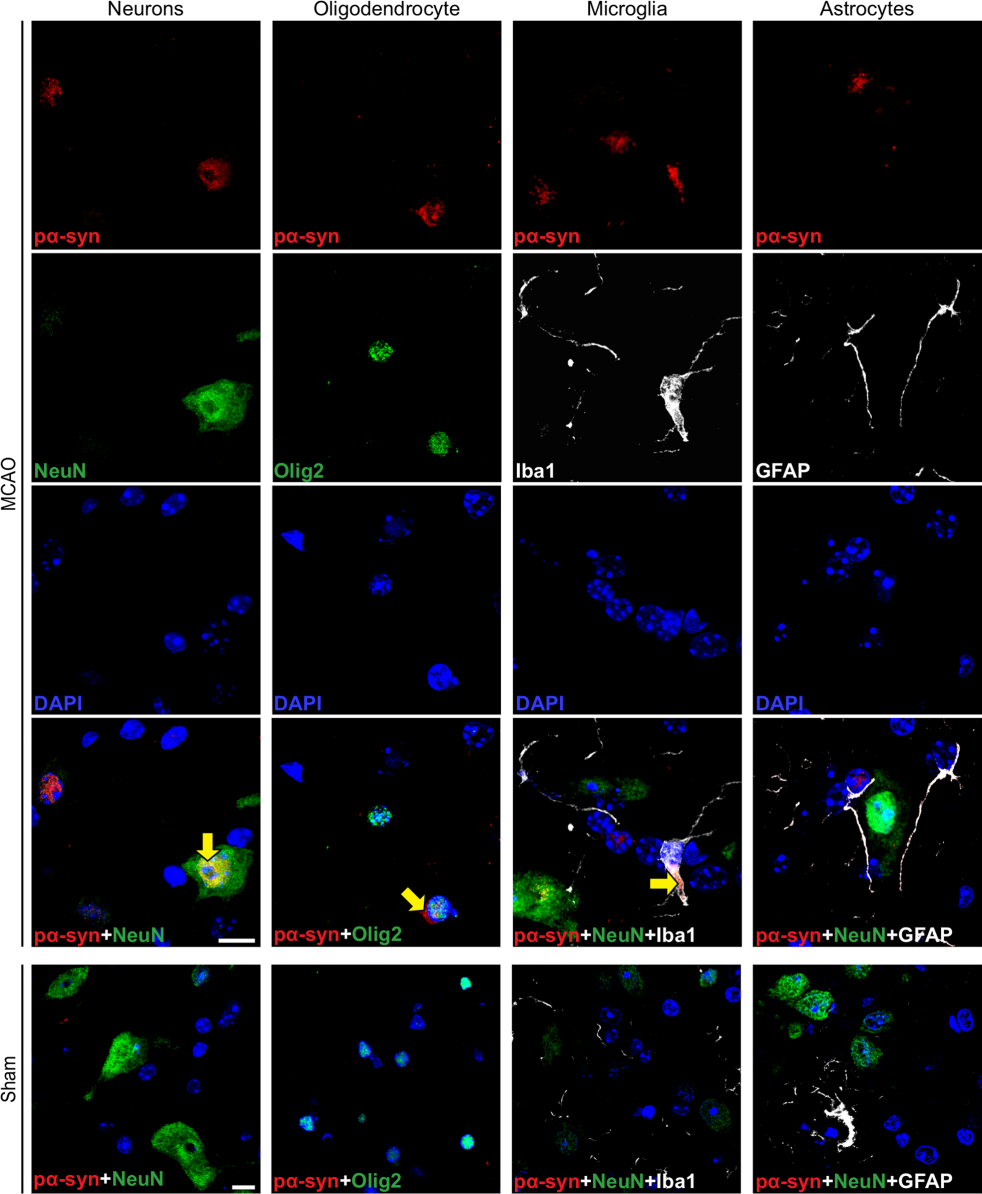
Pathologic α-synuclein colocalizes with neurons, oligodendrocytes, and microglia in the CNS of diseased TgM83^+/−^ mice. Immunofluorescence staining of tissue sections of the brain stem show that phosphorylated α-synuclein (pSyn#64, red) colocalizes with neurons (NeuN, green) as well as with oligodendrocytes (Olig2, green) in mice 360 days after MCAO as indicated by yellow arrows. Deposits of phosphorylated α-synuclein (pSyn#64, red) were additionally seen to colocalize with microglia (Iba1, white) but not with astrocytes (GFAP, white) as indicated by yellow arrows. Neurons, oligodendrocytes, microglia, or astrocytes of mice that underwent sham-surgery, for which only merged images are shown, did not accumulate any phosphorylated α-synuclein deposits. Nuclear staining with DAPI is shown in blue. The scale bar represents 10 µm

To identify which cell types accumulate pathologic α-synuclein, we performed immunofluorescence staining of brain stem tissue sections of mice sacrificed 360 days after MCAO (Fig. 8). Co-staining for phosphorylated α-synuclein and the neuronal marker NeuN revealed neuronal accumulation of pathologic α-synuclein deposits. Co-staining with an antibody to oligodendrocyte transcription factor 2 (Olig2), a marker for oligodendrocytes, showed that also some oligodendrocytes harbored pathologic α-synuclein. Positive co-staining for phosphorylated α-synuclein and the microglial marker Iba1 but not the astrocyte marker GFAP suggests that microglia but not astrocytes phagocytose pathologic α-synuclein from the periphery. In agreement with our previous findings, no deposits of phosphorylated α-synuclein were detected in any of these four cell types in brain stem tissue sections of mice sacrificed 360 days post sham surgery (Fig. 8).

## Discussion

**Fig. 9:**
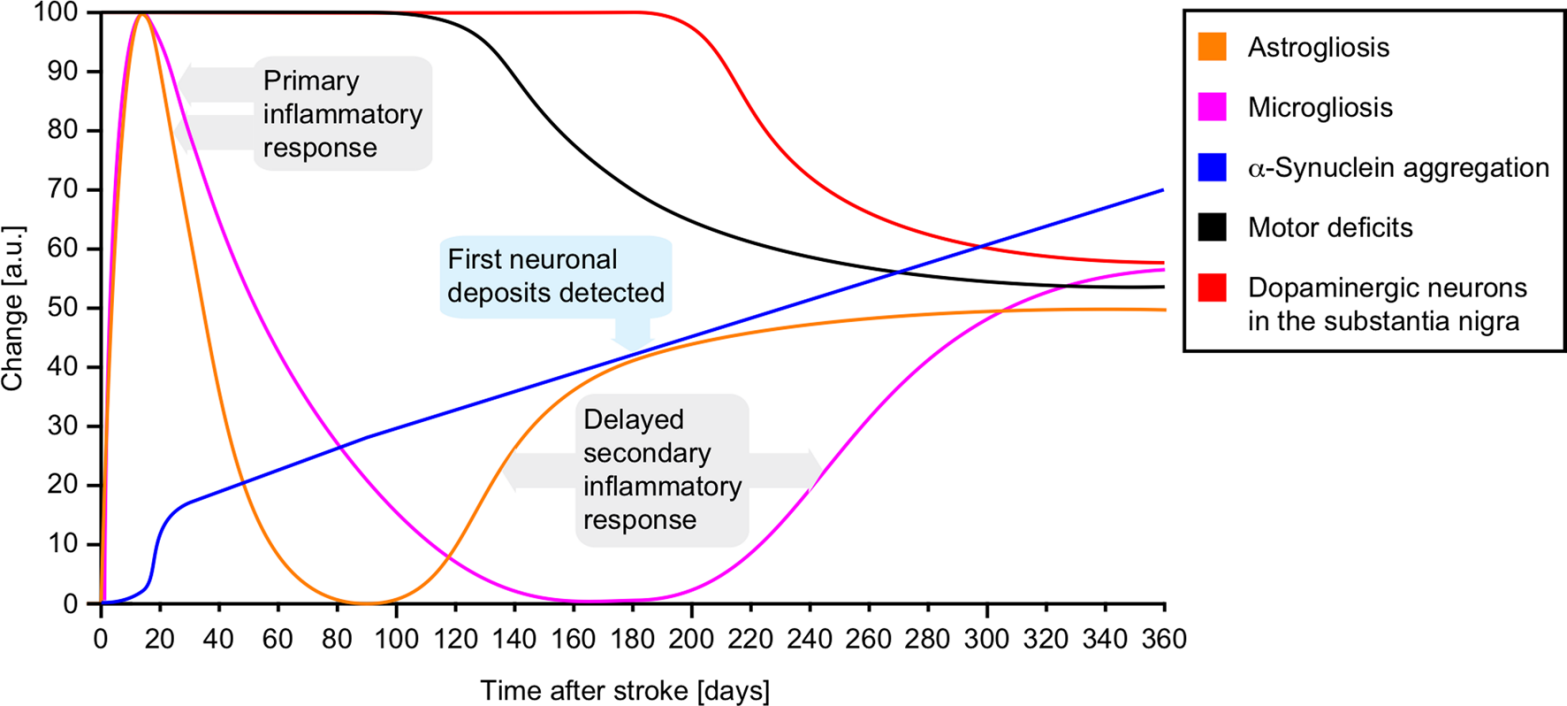
Timeline of pathogenesis in the CNS of TgM83^+/−^ mice after ischemic stroke. Ischemia-induced astrogliosis (orange line) and microgliosis (pink line) both peaked at 14 days after the initial insult, which is also when α-synuclein aggregation (blue line) steadily began to increase based on FRET analysis. Astrogliosis and microgliosis returned close to their pre-ischemic levels by 90 and 180 days post ischemia, respectively, while α-synuclein aggregation continued to increase. Possibly due to the gradually increasing load and deposition of aggregated α-synuclein in the brain, a second inflammatory process set in with a renewed increase in astrogliosis that was slowly followed by microgliosis. By 180 days post ischemia, when cellular deposits of pathologic α-synuclein were first detected, animals also showed first symptoms of motor disease (black line), which worsened over time. By 360 days post ischemia a loss of dopaminergic neurons was detected in the ipsilateral side of the substantia nigra (red line)

Little is known about α-synuclein levels in the brains of patients after stroke. α-Synuclein is naturally present in red blood cells and elevated levels of oligomeric, hemoglobin-bound, and phosphorylated α-synuclein have been reported in red blood cells of ischemic stroke patients relative to healthy subjects [5, 58, 60]. Here we examined the pathologic effects of cerebral ischemia in TgM83^**+/−**^ mice for up to a year after the initial insult (Fig. 9). To our knowledge this is the first study of its kind to investigate the effects of cerebral ischemia on α-synuclein misfolding and its consequences in rodents for such a long duration [25, 53]. Ischemic mice developed significant motor deficits beginning at 180 days post ischemia that worsened over time. Histological analysis revealed that these motor deficits were accompanied by a significant loss of dopaminergic neurons on the ipsilateral side of the substantia nigra, which became evident at 360 days post ischemia. Following MCAO, we also measured a steadily increasing amount of oligomerized/aggregated α-synuclein in the brains of ischemic animals, which resulted in neuronal deposits of pathologic α-synuclein throughout the brain when the animals became symptomatic between 180 and 360 days after ischemia. We also observed a biphasic neuroinflammatory process with microgliosis and astrogliosis in the brains of ischemic animals that was stronger in the ipsilateral side but with time also increasingly present in the contralateral brain hemisphere. Neuroinflammation was first induced directly after MCAO due to the infarct and then gradually subsided. Ischemia and reperfusion are well known to induce an inflammatory response, which enhances clearing mechanisms such as protein degradation [15, 41]. Normally, an inflammatory response eventually subsides, since a continued response would be detrimental to the surrounding tissue. To our surprise, after the initial inflammatory response had subsided a renewed response followed the first and manifested 180–360 days after ischemia. This concomitantly happened during a phase when cellular deposits of pathologic α-synuclein and motor deficits first became detectable. This suggests that the secondary inflammation was in response to neuronal deposition of pathologic α-synuclein and an associated cell loss, which we have previously observed in TgM83^**+/−**^ mice after challenge with synthetic α-synuclein fibrils [10, 32]. Previous studies in wild-type and transgenic mice overexpressing human α-synuclein with the familial A30P mutation subjected to a 30 min MCAO have reported α-synuclein aggregation in ischemic neurons after 72 h of reperfusion [53]. Another study reported aggregation of α-synuclein in ischemic neurons of wild-type mice after a 90 min MCAO with a reperfusion time of four months [25]. Importantly, neither of these studies investigated the long-term effects of stroke on α-synuclein aggregation, the appearance of pathologic α-synuclein in non-ischemic brain regions, nor did they report any loss of dopaminergic neurons or subsequent motor disease in ischemic animals. Our results show that ischemic stroke can trigger α-synuclein aggregation in the CNS and neurological disease in TgM83^**+/−**^ mice long before these animals naturally develop any neuropathology, providing a mechanistic link between cerebral ischemia and the elevated risk of PD.

After ischemia, neurons of TgM83^**+/−**^ mice accumulated cytosolic, dendritic, and perinuclear deposits of pathologic α-synuclein as has been reported for TgM83^**+/−**^ mice challenged with synthetic α-synuclein fibrils or brain homogenates of patients with multiple system atrophy (MSA) [10, 29, 32, 57]. Interestingly, we also detected phosphorylated α-synuclein in the nucleus of some neurons and oligodendrocytes after ischemia. Consistent with our findings, Kim and colleagues also reported a significant increase of phosphorylated α-synuclein in the nucleus of neurons 24 h after ischemia [25]. Deposits of pathologic α-synuclein in oligodendrocytes are characteristic for patients with MSA, where they are mostly localized to the cytosol [47]. Further research is necessary to see whether the ischemic microenvironment might lead to distinct α-synuclein strains that can trigger different types of synucleinopathies [29, 40, 50].

While we find evidence for a pathological cascade that is set in motion after stroke causing α-synuclein to aggregate and resulting in a loss of motor neurons and PD-like symptoms, this study has limitations. We do not know how stroke triggers α-synuclein aggregation. Several possible explanations exist. During MCAO and the following reperfusion, immune cells are activated and recruited to the infarct region and release reactive oxygen species, which have been reported to enhance accumulation and spreading of pathologic α-synuclein in the brain [18, 37]. N- and C-terminal oxidation of methionine residues in α-synuclein by reactive oxygen species is thought to drive α-synuclein oligomerization and inclusion body formation [12, 36]. Also, oxidation of α-synuclein may induce alterations in its structure that inhibit its degradation via the 20S proteasome and thus favor its aggregation [2, 35]. In the aftermath of ischemic stroke, we measured an increase in α-synuclein oligomerization/aggregation in both brain hemispheres. In patients with PD, inflammation and microglial activation in graft deposits is observed long before the accumulation of α-synuclein pathology in implanted dopamine neurons, suggesting that microglial activation contributes to the development of α-synuclein pathology [39]. Our findings suggest that early ischemic events, possibly the spread of reactive oxygen species, may trigger oligomerization/aggregation of α-synuclein in both brain hemispheres, and that a subsequent microglial activation contributes to the neuronal deposition of pathologic α-synuclein. Pathologic α-synuclein may also spread on its own accord to distal brain regions, including the contralateral brain hemisphere, or may be locally induced there to aggregate after the arrival of reactive oxygen species originating in the infarct region [7, 9, 27, 31, 34]. Another limitation is that we decided to terminate this study at 360 days after stroke, when the animals had already developed motor deficits but had not started to lose body weight or to show other overt disease symptoms such as kyphosis yet. Previous studies after challenge with α-synuclein fibrils have shown that TgM83^**+/−**^ mice with terminal disease lose body weight and display more α-synuclein pathology in their CNS than observed here at 360 days after stroke [10, 32]. Another limitation of this study is the lack of wild-type controls. Here we used only TgM83^**+/−**^ mice to investigate the emergence and propagation of pathologic α-synuclein after stroke. Future experiments with possibly longer occlusion times of the middle cerebral artery and/or longer incubation periods after stroke are necessary to conclude whether ischemic stroke can induce a synucleinopathy with clinical symptoms also in wild-type mice.

Few large-scale epidemiological studies have evaluated the association between prior stroke and PD [6, 28]. As a first, Becker and colleagues quantified the risk of a first-time diagnosis of idiopathic PD in patients with a history of stroke using the UK-based General Practice Research Database to compare the prevalence of stroke/TIA in 3637 newly diagnosed PD patients and in a matched comparison group without PD between 1994 and 2005 [6]. They concluded that a history of stroke was associated with a significantly increased relative risk of being diagnosed with PD compared to patients without such a history with an adjusted odds ratio of 1.65 and a 95% confidence interval of 1.47–2.00. More recently, Kummer and colleagues conducted a much larger retrospective cohort study using claims data from a 5% random sample of Medicare beneficiaries in the USA from 2008–2015 [28]. Among 1,035,536 Medicare beneficiaries followed for a mean of 5.2 years, 15,531 (1.5%) participants were diagnosed with PD. In this study, stroke was one of the highest risk factors associated with a subsequent diagnosis of PD with a hazard ratio of 1.55 and a 95% confidence interval of 1.39–1.72. Both studies conclude that ischemic stroke increases the risk of developing PD. More importantly, because these studies do not evaluate the risk of PD after silent stroke, which affect up to 28% of the population, theses studies likely underestimate the risk of PD after stroke [54, 55].

To date no mechanistic insights or animal models exist that sufficiently explain this causal relationship or could be used to study it. Here, we have established a new mouse model to investigate this problem: MCAO in TgM83^**+/−**^ mice in combination with a one-year follow up revealed a causal link between mild cerebral ischemia and PD. Cerebral ischemia resulted in aggregation of α-synuclein and a concomitant loss of dopaminergic neurons in the substantia nigra, which subsequently resulted in PD-like symptoms. Mice displayed significant motor deficits within a year after MCAO. Neuroinflammation was first significantly induced at an early time point due to the infarct and then quickly subsided and, surprisingly, resurfaced again after 6–12 months. This late-stage neuroinflammatory response coincided with a significantly increased deposition of pathologic α-synuclein throughout the brain. Our findings provide a possible explanation of how cerebral ischemia can lead to PD and an animal model to further study this relationship and potential therapeutic interventions.

## Supplementary Information

**Additional file 1: Table S1**. Antibodies used for immunofluorescence and immunohistochemistry.

**Additional file 2**. TgM83^+/−^ mice do not show any neuronal loss following sham surgery Immunohistochemical staining for NeuN showed that sham surgery did not cause any neuronal loss in the ipsilateral or contralateral brain hemispheres of TgM83^+/−^ mice at 14 (**a**), 30 (**b**), 90 (**b**), 180 (**b**), or 360 days (**b**) after sham surgery. The scale bar represents 0.5 mm.

**Additional file 3**. Spread of neuroinflammation to the contralateral brain hemisphere by 360 days after stroke. Staining of brain tissue sections of MCAO- and sham-treated TgM83^+/−^ mice with antibodies to Iba1 (**a**) and to GFAP (**b**) revealed that microgliosis and astrogliosis had spread to the contralateral brain hemisphere by 360 days after stroke. A higher amount of microglia was found throughout the entire contralateral brain hemisphere of MCAO-treated animals compared to sham-treated animals (**a**). Also, a higher amount of astrocytes was detected in the contralateral brain hemisphere of MCAO-treated animals compared to sham-treated animals, especially in the lower isocortical region and the upper part of the caudoputamen (**b**). Scale bars represent 1.0 mm.

**Additional file 4**. Quantification of phosphorylated α-synuclein deposits in different brain regions of TgM83^+/−^ at 360 days after stroke. The number of phosphorylated α-synuclein-positive cells per square millimeter was plotted for the ipsilateral and contralateral brain hemisphere for each quantified brain area of mice subjected to stroke. The coordinates of the quantified coronal tissue sections relative to the bregma were 0.74 mm (**a**), −1.70 mm (**b**), −2.92 mm (**c**), −4.84 mm (**d**), and −5.68 mm (**e**) as shown in the heat map in Fig. 7b. Data shown represent the mean ± standard deviation of four animals.

**Additional file 5**. TgM83^+/−^ mice harbor deposits of pathologic α-synuclein throughout the brain at 180 days after stroke. Immunohistochemical staining of coronal brain tissue sections with the pSyn#64 antibody against α-synuclein phosphorylated at serine 129 revealed deposits throughout the brain of diseased animals at 180 days after MCAO. Deposits were present throughout the cerebrum, including the thalamus, hypothalamus, substantia nigra, and superior colliculus. In contrast, none of the animals that underwent sham surgery displayed any deposits of phosphorylated α-synuclein, even at 360 days after surgery. The scale bar represents 10 μm.

## Data Availability

All data generated or analyzed during this study are included in this published article [and its supplementary information files].
