# Supplementary material for: Ischemic stroke causes Parkinson’s disease-like pathology and symptoms in transgenic mice overexpressing alpha-synuclein
Source: Acta Neuropathol Commun. 2022 Feb 24;10:26. doi: 10.1186/s40478-022-01327-6 (PMC8867857; doi:10.1186/s40478-022-01327-6)
Supplement: Supplementary file 1 — Additional file 1: Table S1. Antibodies used for immunofluorescence and immunohistochemistry. [file 40478_2022_1327_MOESM1_ESM.pdf]

Table S1 Antibodies used for immunofluorescence and immunohistochemistry

Target (alternative name) [antibody clone]	Source	Catalogue number	Host	Dilutions for IF	Dilutions for IHC	Buffers for antigen retrieval
alpha-Synuclein (phospho S129) [81A]	Covance	MMS-5091	Mouse	–	1:200	Citrate buffer
alpha-Synuclein (phospho S129) [EP1536Y]	Abcam	AB51253	Rabbit	–	1:500,000	–
alpha-Synuclein (phospho S129) [pSyn#64]	Wako	015-25191	Mouse	1:1200	–	Citrate buffer
alpha-Synuclein (phospho S129) [pSyn#64], biotin-conjugated	Wako	010-26481	Mouse	–	1:1000	Formic acid
Glial fibrillary acidic protein (GFAP)	Dako	Z0334	Rabbit	–	1:500	Formic acid
Glial fibrillary acidic protein (GFAP)	Invitrogen	13-0300	Rat	1:150	–	Citrate buffer
Ionized calcium binding adaptor molecule 1 (Iba1)	Wako	019-19741	Rabbit	–	1:500	Formic acid
Ionized calcium binding adaptor molecule 1 (Iba1)	Synaptic Systems	234004	Guinea pig	1:400	–	Citrate buffer
Neuronal nuclei (NeuN) [A60]	Merck Millipore	MAB377	Mouse	–	1:1000	Citrate buffer

Neuronal nuclei (NeuN) [clone 27-4]	Merck Millipore	MABN140	Rabbit	1:1000	–	Citrate buffer
Oligodendrocyte transcription factor 2 (Olig2)	Merck Millipore	AB9610	Rabbit	1:500	–	Citrate buffer
Tyrosine hydroxylase (TH)	Merck Millipore	ab152	Rabbit	–	1:1000	Citrate buffer

IF, immunofluorescence staining; IHC, immunohistochemistry
