# Supplementary figures and images for: Ischemic stroke causes Parkinson’s disease-like pathology and symptoms in transgenic mice overexpressing alpha-synuclein
Source: Acta Neuropathol Commun. 2022 Feb 24;10:26. doi: 10.1186/s40478-022-01327-6 (PMC8867857; doi:10.1186/s40478-022-01327-6)

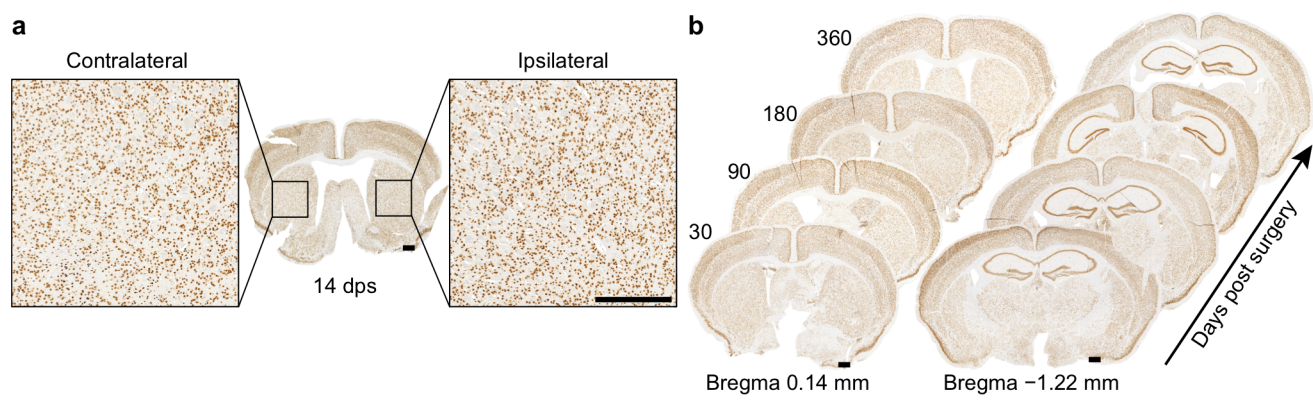

Supplement: Supplementary file 2 — Additional file 2. TgM83+/− mice do not show any neuronal loss following sham surgery Immunohistochemical staining for NeuN showed that sham surgery did not cause any neuronal loss in the ipsilateral or contralateral brain hemispheres of TgM83+/− mice at 14 (a), 30 (b), 90 (b), 180 (b), or 360 days (b) after sham surgery. The scale bar represents 0.5 mm. [file 40478_2022_1327_MOESM2_ESM.pdf]

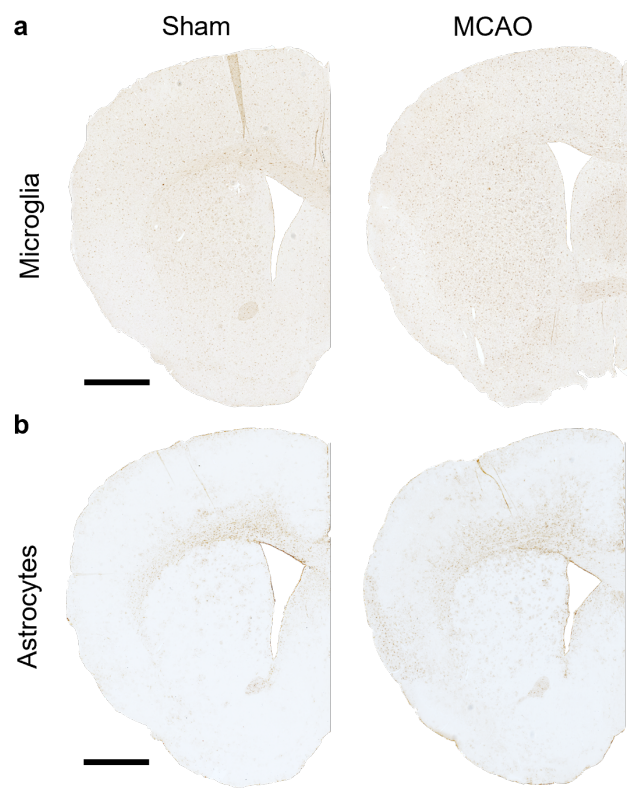

Supplement: Supplementary file 3 — Additional file 3. Spread of neuroinflammation to the contralateral brain hemisphere by 360 days after stroke. Staining of brain tissue sections of MCAO- and sham-treated TgM83+/− mice with antibodies to Iba1 (a) and to GFAP (b) revealed that microgliosis and astrogliosis had spread to the contralateral brain hemisphere by 360 days after stroke. A higher amount of microglia was found throughout the entire contralateral brain hemisphere of MCAO-treated animals compared to sham-treated animals (a). Also, a higher amount of astrocytes was detected in the contralateral brain hemisphere of MCAO-treated animals compared to sham-treated animals, especially in the lower isocortical region and the upper part of the caudoputamen (b). Scale bars represent 1.0 mm. [file 40478_2022_1327_MOESM3_ESM.pdf]

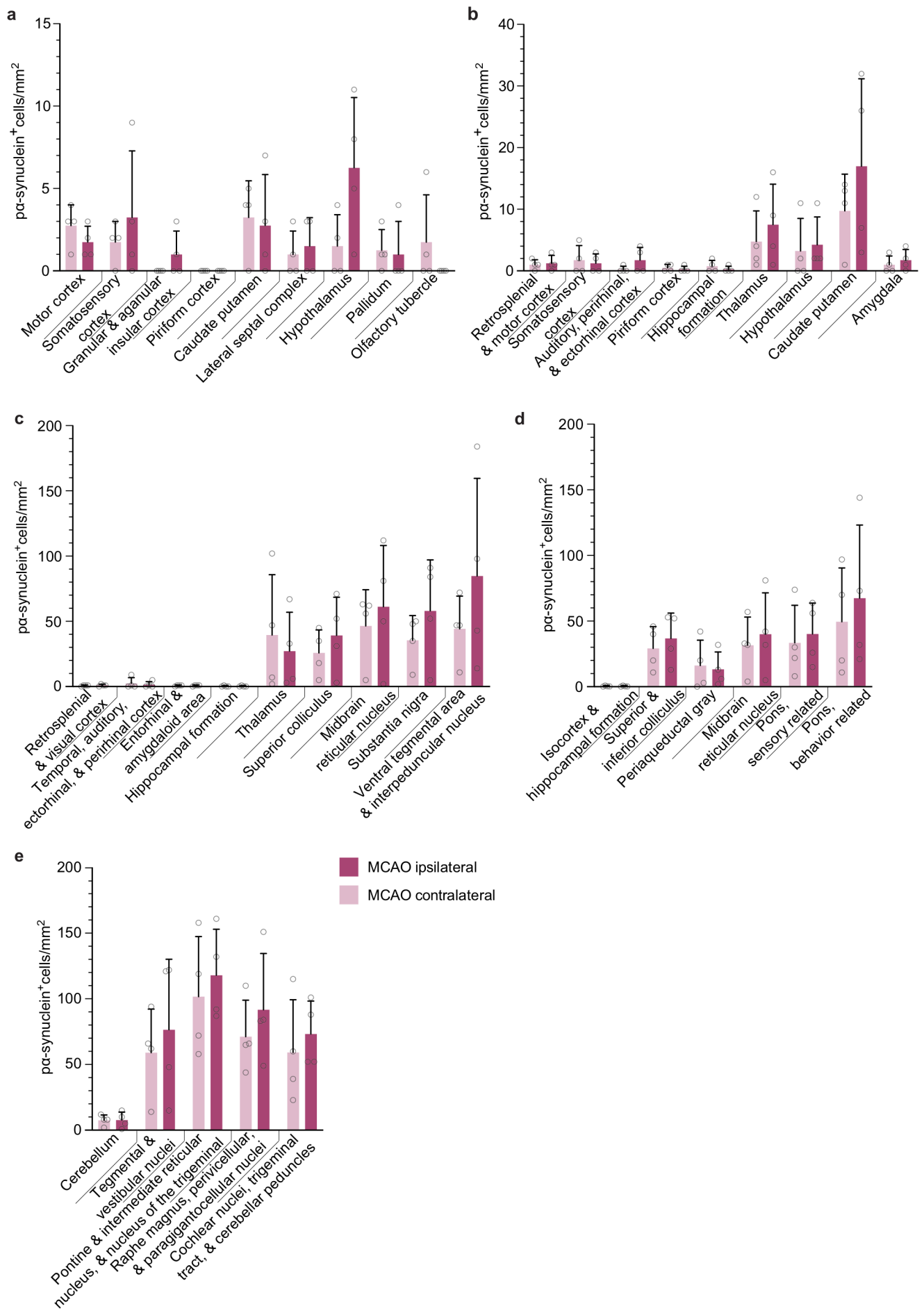

Supplement: Supplementary file 4 — Additional file 4. Quantification of phosphorylated α-synuclein deposits in different brain regions of TgM83+/− at 360 days after stroke. The number of phosphorylated α-synuclein-positive cells per square millimeter was plotted for the ipsilateral and contralateral brain hemisphere for each quantified brain area of mice subjected to stroke. The coordinates of the quantified coronal tissue sections relative to the bregma were 0.74 mm (a), −1.70 mm (b), −2.92 mm (c), −4.84 mm (d), and −5.68 mm (e) as shown in the heat map in Fig. 7b. Data shown represent the mean ± standard deviation of four animals. [file 40478_2022_1327_MOESM4_ESM.pdf]

Thalamus

Hypothalamus

Substantia nigra

Superior colliculus

MCAO 180 dps

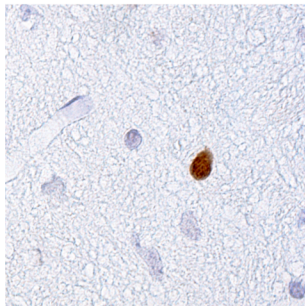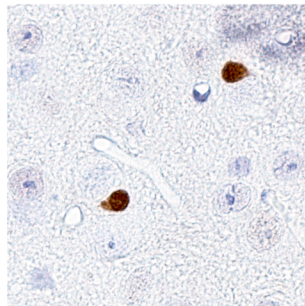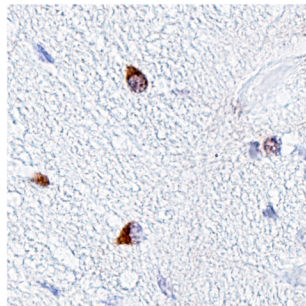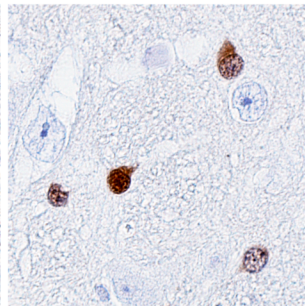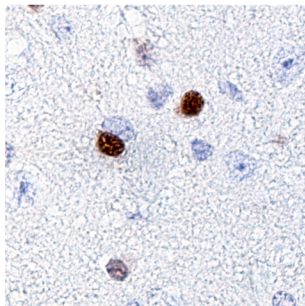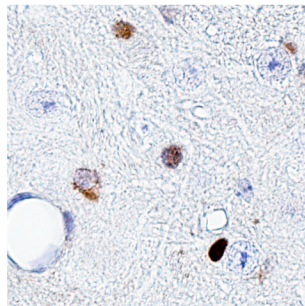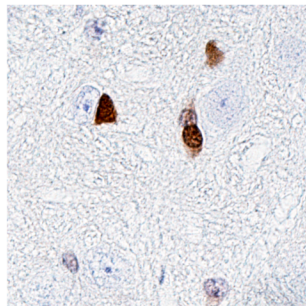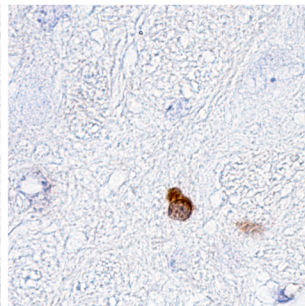

Sham 360 dps

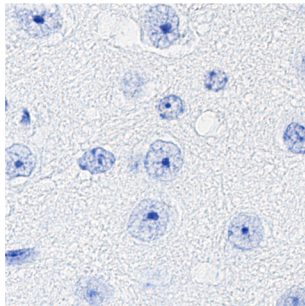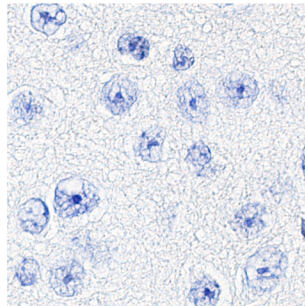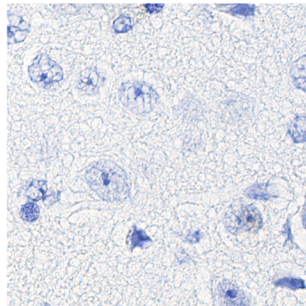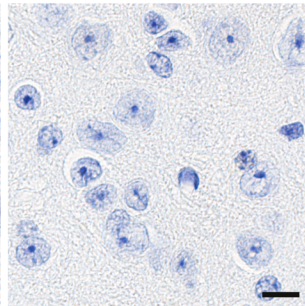

Supplement: Supplementary file 5 — Additional file 5. TgM83+/− mice harbor deposits of pathologic α-synuclein throughout the brain at 180 days after stroke. Immunohistochemical staining of coronal brain tissue sections with the pSyn#64 antibody against α-synuclein phosphorylated at serine 129 revealed deposits throughout the brain of diseased animals at 180 days after MCAO. Deposits were present throughout the cerebrum, including the thalamus, hypothalamus, substantia nigra, and superior colliculus. In contrast, none of the animals that underwent sham surgery displayed any deposits of phosphorylated α-synuclein, even at 360 days after surgery. The scale bar represents 10 μm. [file 40478_2022_1327_MOESM5_ESM.pdf]
